# COVID-19 and benign intracranial hypertension: A case report

**DOI:** 10.1590/0037-8682-0325-2020

**Published:** 2020-06-08

**Authors:** Fabio Noro, Fernando de Mendonça Cardoso, Edson Marchiori

**Affiliations:** 1 Universidade Federal do Rio de Janeiro, Departamento de Radiologia, Rio de Janeiro, RJ, Brasil; 2 Hospital Copa D’Or, Rede D’Or São Luiz, Serviço de Neurologia, Rio de Janeiro, RJ, Brasil.

A 35-year-old female patient, without comorbidities, presented to the emergency room with fever, dyspnea, and adynamia over the previous four days. She also had a complaint of a headache that had started two days before admission. She was lucid and oriented, without focal neurological deficits. During hospitalization, the headache worsened and she became disoriented. Fundoscopy was impaired. A cerebrospinal fluid puncture was performed, showing increased pressure (40 cm H2O). The cerebrospinal fluid analysis was entirely normal and negative for several pathogens. Real-time reverse-transcription polymerase chain reaction testing of a nasopharyngeal swab confirmed SARS-Cov-2 infection.

**FIGURE 1:**
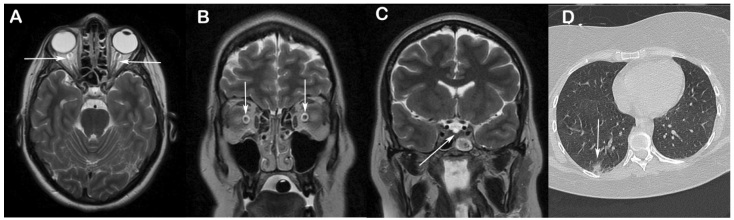
**(A and B)** T2 axial and coronal magnetic resonance imaging scan shows prominent subarachnoid space around the optic nerves and their vertical tortuosity (arrows); **(C)** Coronal T2 magnetic resonance imaging scan of the selar region demonstrates compression of the upper portion of the pituitary gland by the selar diaphragm (arrow). **(D)** Chest computed tomography shows round peripheral ground-glass opacities in the right lung (arrow).

A brain magnetic resonance imaging scan showed signs of intracranial hypertension characterized by prominent subarachnoid space around optic nerves, vertical tortuosity of the optic nerves, and superior compression of the hypophysis (Figure 1A-C). Chest computed tomography showed round ground-glass opacities in both lungs (Figure 1D). The final diagnosis, based on clinical, laboratory, and imaging findings was benign intracranial hypertension (BIH).

The patient was treated with supportive measures and nasal oxygen. After two days of hospitalization, the patient returned to a normal level of consciousness and the headache disappeared. She was discharged asymptomatic two days later.

BIH (pseudotumor cerebri) is a clinical condition that includes headache, papilledema, increased blood pressure, and clear cerebrospinal fluid^1^. Multiple causes have been described for this condition, including venous sinus thrombosis, the toxicity of some substances such as vitamin A, tetracyclines and contraceptives, and sepsis^1^. To our knowledge, this is the first report of COVID-19 associated with isolated BIH.
